# Stress Cardiomyopathy (Takotsubo syndrome) Following Accidental Methadone Poisoning; Report of Two Pediatric Cases

**Published:** 2019-03-09

**Authors:** Khatereh Dehghani, Mohammad Shojaie, Amir Hossein Pourdavood, Mohammad Khajouei

**Affiliations:** 1 Department of Cardiology, Jahrom University of Medical Sciences, Jahrom, Iran.; 2 Cardiology Department, Non-communicable Research Center, Jahrom University of Medical Sciences, Jahrom, Iran; 3 Department of Surgery, Kerman University of Medical Sciences, Kerman, Iran.; 4 Nanotechnology Research Institute, Chemical Engineering Department, Babol (Noshirvani) University of Technology, Babol, Iran.

**Keywords:** Methadone, toxicity, cardiomyopathy, pediatrics, case reports

## Abstract

Methadone poisoning has become more common in the pediatric population due to extensive use of methadone maintenance therapy (MMT). It is associated with decreased level of consciousness, coma, respiratory distress and cardiac intoxication. The cardiac complications have been reported to be QT prolongation, torsade de pointes, coronary artery disease, arrhythmia, stress cardiomyopathy and death. We herein report two pediatric patients with accidental methadone poisoning who developed stress cardiomyopathy and cardiac failure. The first case was a 4-yaer-old girl and the second one was an 18-month-old girl both being accidentally poisoned with methadone syrup and were brought with decreased level of consciousness. Both were diagnosed to suffer from congestive heart failure based on echocardiography. However, the first case passed away despite appropriate treatment, while the second one survived the condition and was discharged with good condition and was symptom free at 6-month follow-up.

## Introduction

Methadone is a synthetic opioid which was first introduced in Germany between 1937 and 1939. A long half-life of 25 to 52 hours and high analgesic effects, makes methadone suitable for maintenance therapy in opium addicts and is currently used as the basis of withdrawal management as methadone maintenance therapy (MMT) (1). Methadone poisoning is among the catastrophic intoxications with a wide range of clinical presentations including decrease in the level of consciousness, coma, apnea, respiratory suppression, and death (2). In recent years, a significant rise in the use of different forms of methadone (tablet and syrup) in Iran has led to increase in reported rates of accidental poisonings especially in pediatric populations (3, 4). Methadone overdose in children has been associated with respiratory distress, cardiac toxicity and decreased level of consciousness. It has also been associated with nausea and vomiting, myosis, dyspnea and early seizures (5, 6). Respiratory suppression has been reported to be the most lethal complication of methadone intoxication, which is initialized approximately 12 hours after drug ingestion (7). A common cardiac manifestation in methadone overdose is QT prolongation, which can lead to torsade de pointes and may result in death (8). Other effects include changes in QT dispersion, pathological U waves, stress cardiomyopathy (Takotsubo syndrome), Brugada-like syndrome, and coronary artery diseases (9). We herein report, two cases of stress cardiomyopathy in pediatrics following methadone poisoning. 

## Case presentation

***Case 1***

**Figure 1:**
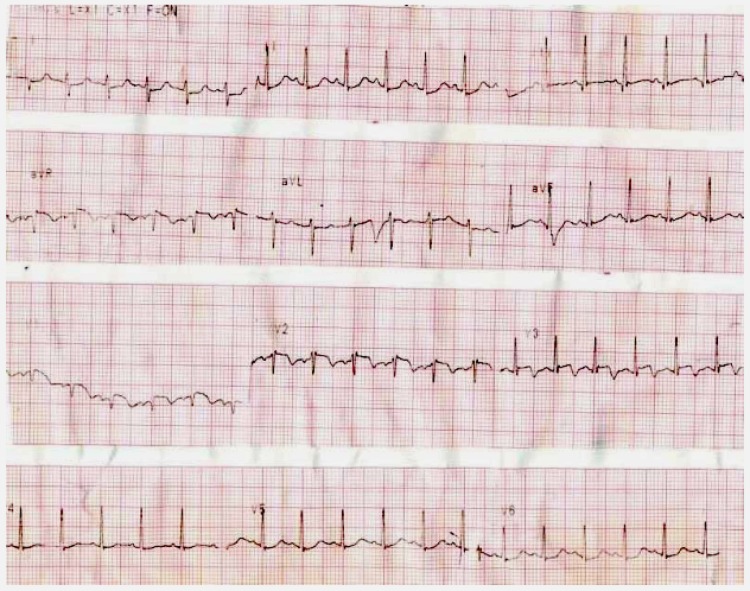
The 12-lead electrocardogram (ECG) of the first patient demonstrating tachycardia with QT interval of 440 milliseconds and ST segment elevation in aVR and T wave inversion V1, V2 and V3

A 4-year-old girl was brought to our emergency department with decreased level of consciousness since an hour before. She was the second child of the family whose father was an opium addict on MMT. The methadone had been kept in a bottle like pediatric syrup bottle and the girl was mistakenly given methadone instead of cough syrup by her mother. Upon arrival she had a Glasgow coma score (GCS) of 7/15, a heart rate of 145 bpm, a respiratory rate of 32 breaths/minute, blood pressure of 132/76 mmHg and a temperature of 36.8°C with an oxygen saturation of 85% in room air. Her pupils were myotic and responsive to light and other examinations were within normal limits, so treatment by naloxone (0.1 mg/kg) was started for her. Since there was no response to the initial therapy, she was admitted to the intensive care unit (ICU) and maintenance naloxone (0.16 mg/kg/h) was started. Her electrocardiogram showed tachycardia with QT interval of 440 milliseconds and ST segment elevation in aVR and T wave inversion V1, V2 and V3 (Figure 1). Laboratory investigations revealed white blood cell (WBC) count of 31,800 /mm3, hemoglobin of 11.9 mg/dL, platelet count of 291,000/mm3, blood urea nitrogen (BUN) of 24 mg/dL, creatinine (Cr) of 1.7 mg/dL, sodium of 133 mEq/L, potassium of 6 mEq/L, blood sugar of 113 mg/dL, calcium of 8.9 mg/dl, creatine phosphokinase (CPK) of 32,000 IU/L, lactate dehydrogenase (LDH) of 6,030 IU/L, troponin of 6.18 U/L, prothrombin time (PT) of 24.4 sec, partial thromboplastin time (PTT) of 48 second, SGOT of 123 U/L and SGPT of 1545 U/L. 

Transthoracic echocardiography revealed left ventricular ejection fraction of 25% with akinesia of posterior wall and hypokinesia of septum. She was diagnosed with stress cardiomyopathy and acute heart failure and thus the management of heart failure including diuretics and inotropes was stared for her. On the second day of admission, she developed respiratory arrest and thus was intubated and connected to the ventilator with GCS of 3/15. Brain CT-scan showed bilateral cerebellar infarction and edema and diffuse brain edema. Neurologist was consulted and medical management of intracranial hypertension was started. The patient received hypertonic saline (5mg/kg as bolus dosage and 3mg/kg every 6 hours) and mannitol (1 mg/kg/dose). However, unfortunately she died on the third day despite intensive care management.

***Case 2***

**Figure 2:**
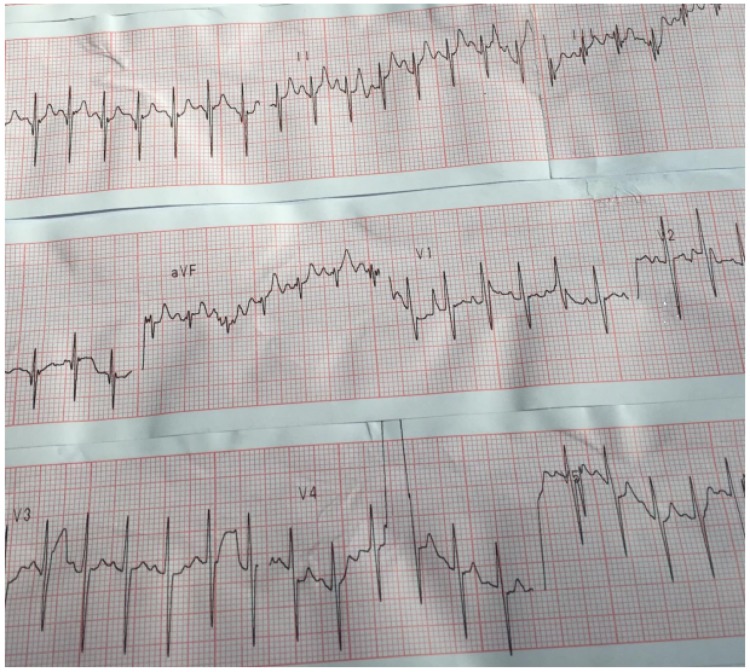
The 12-lead electrocardogram (ECG) of the second patient demonstrating sinus tachycardia and prolonged QT-interval

An 18-month-girl was transferred to our center from a primary healthcare center due to decreased level of consciousness several hours prior to presentation. She was reported to be treated with 3cc of methadone solution by mistake instead of diphenhydramine because of coryza signs and symptoms. On admission to the primary center, she had a GCS of 14/15, a heart rate of 135 bpm, a respiratory rate of 26 breaths/minute, blood pressure of 127/67 mmHg and a temperature of 36.8°C with an oxygen saturation of 98% in room air. Her pupils were myotic with response to the light. With the primary impression of methadone poisoning, naloxone (0.1 mg/kg) had been administered in the primary center and then she was transferred here. She was also reported to have had 2 episodes of tonic-clonic convulsions in the ambulance during the transfer for which diazepam (0.1 mg/kg) was administered. On admission to our center, she had GCS of 8/15 and she had bradypnea with abdominal respiration and O_2_ saturation of 84% without oxygen supplement, blood pressure of 117/67 mmHg, a temperature of 37.5°C with myotic pupils that were reactive to light and upward plantar reflex (Babinski), so naloxone infusion was continued (0.16 mg/kg/h). She was intubated and transferred to ICU. She had two episodes of tonic clonic convulsion for which midazolam infusion was started (0.05 mg/kg/hr). Laboratory examination revealed WBC count of 18,000/mm3 (88%PMN, 11%Lymphocyte), hemoglobin of 8.6 mg/dL, platelet count of 390,000/mm3, BUN of 18 mg/dL, Cr of 0.7 mg/dL, sodium of 140 mEq/mL, potassium of 4.7 mEq/L, blood sugar of 102 mg/dL, CPK of 3,050 IU/L, LDH of 2,375 IU/L, PT of 20 second, PTT of 28 second, SGOT of 6220 U/L, SGPT of 5740 U/L and CRP of 73 mg/L. The ECG also showed sinus tachycardia and prolonged QT interval (Figure 2). On the second day, she had another episode of convulsion with decrease in O_2_ saturation. She developed bilateral fine rales in lung examination in favor of pulmonary edema, so infusion of furosemide (1 mg/kg q2h) was started. Chest radiography revealed pulmonary edema. Brain CT scanning demonstrated a small area of ischemia and hydrocephaly. 

In echocardiography, ejection fraction of 15% with diffuse hypokinesia and mild mitral valves regurgitation has been showed. She was diagnosed to suffer from stress cardiomyopathy for which management of heart failure was started. After 3 days of conservative management with naloxone (0.1 mg/kg/dose) and phenobarbital (0.15 mg/kg q12h) the patient developed spontaneous respiration and was extubated 2 days later. Her GCS increased to 14/15 and echocardiography revealed normal cardiac function. She was discharged from the hospital on the 12^th^ day while she was completely awake with normal echocardiography. On the 6-month follow-up she had normal daily activity and normal echocardiography without signs of cardiac failure. She did not have any seizure so the anticonvulsants were tapered. 

## Discussion:

Methadone is a powerful analgesic drug that affects mu receptors and it is majorly used for treatment of opioid addiction as well as for pain relief (9, 10). Poisoning by methadone is very hazardous and may lead to death because of its irreversible complications; in most countries it is kept in containers that it is not easy for children to open and also warning labels attached on the containers (11). However, in Iran there is not any special bottle for methadone in form of syrup and it is kept in bottles like pediatric syrup bottles, so the rate of pediatric methadone poisoning is relatively high and a large number of hospitalizations in emergency ward are due to this type of poisoning (12). Unfortunately, this drug is sometimes administered to children instead of other pediatric syrups by mistake and because of its silent symptoms such as drowsiness and loss of consciousness, it’s not detected early on by parents and they usually find out several hours after drug ingestion, when it is too late to help these children survive (3, 9). 

Complications of methadone toxicity are drowsiness, decreased level of consciousness, respiratory failure, myosis, hypotension, acute respiratory distress syndrome (ARDS), brain damage and other end organ damages (9). Respiratory suppression is the main cause of death in patients with methadone overdose, it may lead to severe hypoxia that can result in lung and brain injury as well as renal and liver dysfunction (13). Some other adverse effects include ataxia, hearing loss, cerebellitis and chest rigidity (14, 15). The cardiac complications of methadone toxicity are rare and include QT prolongation and subsequent torsade de pointes mainly because of the electrolyte imbalance (9). 

We herein reported two pediatric patients with methadone poisoning who developed severe left ventricular dysfunction and stress cardiomyopathy leading to death in one. Stress cardiomyopathy (Tako-Tsubo syndrome) is defined as left ventricular dysfunction and ECG changes that mimic acute myocardial infarction without any evidence of involvement of coronary arteries (16, 17). It is more common in postmenopausal women and an acute medical illness or emotional/physical stress may typically trigger this syndrome. It has been demonstrated that huge release of catecholamines (maybe due to opioid withdrawal) may be responsible for development of this cardiomyopathy. Two cases of Taku-Tsubo syndrome have been reported in relation with acute opioid withdrawal. Lemesle et al. (16), reported the first case as a result of methadone withdrawal secondary to drug-drug interaction. However, we observed this phenomenon in 2 pediatric cases who were admitted to our center with methadone poisoning and the mortality rate was as high as 50% in our patients. All the previously reported cases were adults but our patients were children. Two cases of Taku-Tsubo cardiomyopathy have been reported previously, but both of them had happened after methadone withdrawal not methadone poisoning (16, 17). In our patients, autopsy was not done in the first patient to clear the cause of her cardiac damage and the condition of the second one wasn`t suitable for cardiac MRI to evaluate the cardiac structure and function. Further evaluation on other cases is needed to clear the effect of methadone and its metabolites on the myocardium.

## Conclusion:

 Methadone overdose can cause cardiac injury leading to cardiac failure and death. Toxic metabolites of methadone may cause myocarditis and cardiac failure while excessive catecholamine release could be another explanation for this phenomenon especially in young children.
